# Identification of *NF1* Frameshift Variants in Two Chinese Families With Neurofibromatosis Type 1 and Early-Onset Hypertension

**DOI:** 10.3389/fped.2021.785982

**Published:** 2021-12-20

**Authors:** Yi-Ting Lu, Di Zhang, Xin-Chang Liu, Qiong-Yu Zhang, Xue-Qi Dong, Peng Fan, Yan Xiao, Xian-Liang Zhou

**Affiliations:** Department of Cardiology, National Center for Cardiovascular Diseases, Fuwai Hospital, Chinese Academy of Medical Sciences and Peking Union Medical College, Beijing, China

**Keywords:** neurofibromatosis type 1, *NF1*, early-onset hypertension, frame-shift mutation, genetic analysis

## Abstract

**Background:** Neurofibromatosis type 1 (NF-1) is a common autosomal dominant disorder caused by mutations in the *NF1* gene. It is characterized by multiple café-au-lait macules, cutaneous neurofibromas, optic glioma, Lisch nodules, and axillary and inguinal freckling. The aim of this study was to investigate *NF1* mutations in two Chinese families with NF-1 who presented with early-onset hypertension, and to determine the prevalence of hypertension associated with NF-1 to better understand this complication.

**Methods:** Whole-exome sequencing was performed for the probands with NF-1 from two unrelated families. Possible pathogenic mutation was predicted by bioinformatic tools. Sanger sequencing was used to confirm candidate variants in all available individuals for familial co-segregation analysis. We also performed a systematic literature review of studies that reported the prevalence of hypertension in patients with NF-1.

**Results:** In family 1, a recurrent mutation c.6789_6792delTTAC in *NF1* was identified in the proband but in no other family members, indicating that this is a *de novo* mutation. In family 2, a novel mutation c.6934_6936delGCAinsTGCT in *NF1* was detected in the proband and two other family members, which co-segregated with the disease phenotype within the family. Both mutations were predicted to be pathogenic by bioinformatic analysis. We found hypertension was a relatively common complication of NF-1, with a prevalence range of 6.1–23.4%. Ambulatory blood pressure monitoring is a stable method for detecting initial alterations of the blood pressure pattern, particularly for pre-hypertension.

**Conclusions:** We identified one recurrent (c.6789_6792delTTAC) and one novel frame-shift mutation (c.6934_6936delGCAinsTGCT) in two unrelated families with NF-1 using whole-exome sequencing. In consideration of phenotypic heterogeneity in NF-1, genetic testing is a robust tool which helps early and accurate diagnosis. Because hypertension is not a rare complication of NF-1, routine screening for hypertension in patients with NF-1, especially children and adolescents, is important to avoid serious cardiovascular events.

## Introduction

Neurofibromatosis type 1 (NF-1, MIM 162200), also known as von Recklinghausen disease, is a progressive autosomal dominant disorder. NF-1 is one of the most common hereditary diseases with an estimated prevalence of 1 in 2,500 to 1 in 3,000 persons worldwide (1, 2). The disease is caused by mutations in the neurofibromin gene (*NF1*), a tumor suppressor gene mapping to 17q11.2, and spans 60 exons with an approximate size of 350 kb. *NF1* encodes neurofibromin, which belongs to the Ras-guanosine triphosphatase (RAS-GTPase) activating protein family. Loss-of-function mutations in *NF1* lead to intracellular neurofibromin protein deficiency, followed by over-activation of RAS signaling to the downstream pathways, which consequently leads to the development of phenotypes characterized by pigmentary lesions and predispositions to neoplastic disorders (3).

Despite having an early complete penetrant age by 5 years, the clinical expression of NF-1 is highly variable from one individual to another (4). NF-1 affects a wide range of organs, characterized by multiple café-au-lait macules (CALM), cutaneous neurofibromas, optic glioma, Lisch nodules, and axillary and inguinal freckling. Cognitive difficulties, skeletal abnormalities, and mental disorders are also common and the severity of symptoms is closely related to long-term impaired quality of life (5). Because of the phenotypic heterogeneity, timely diagnosis of NF-1 is challenging and genetic analysis is considered a robust tool that can assist with molecular diagnosis and intrafamilial genetic counseling.

Hypertension is not rare in patients with NF-1 (with a prevalence of 16–19% in children), no matter whether it is the essential form or secondary to renal or aortic vasculopathy, or pheochromocytoma (6, 7). Hypertension complications or NF-1 itself are both associated with increased cardio-cerebrovascular risk (8, 9). Routine blood pressure screening is indispensable to effectively detect elevated blood pressure and avoid severe negative effects on targeted organs. The proportion of patients with renovascular hypertension or hypertension caused by pheochromocytoma who also develop NF-1 has been analyzed previously (10, 11). We focused on the prevalence of hypertension and various screen methodology for hypertension in the general NF-1 population.

Here, we investigated two unrelated Chinese families with NF-1 characterized by early-onset hypertension by whole-exome sequencing and Sanger sequencing and identified one recurrent *NF1* variant c.6789_6792delTTAC and one novel heterozygous mutation c.6934_6936delGCAinsTGCT in families 1 and 2, respectively. We also summarize the data from previous clinical studies into the hypertension associated with NF-1 to better understand this complication.

## Materials and Methods

### Subjects

**Figure 1:**
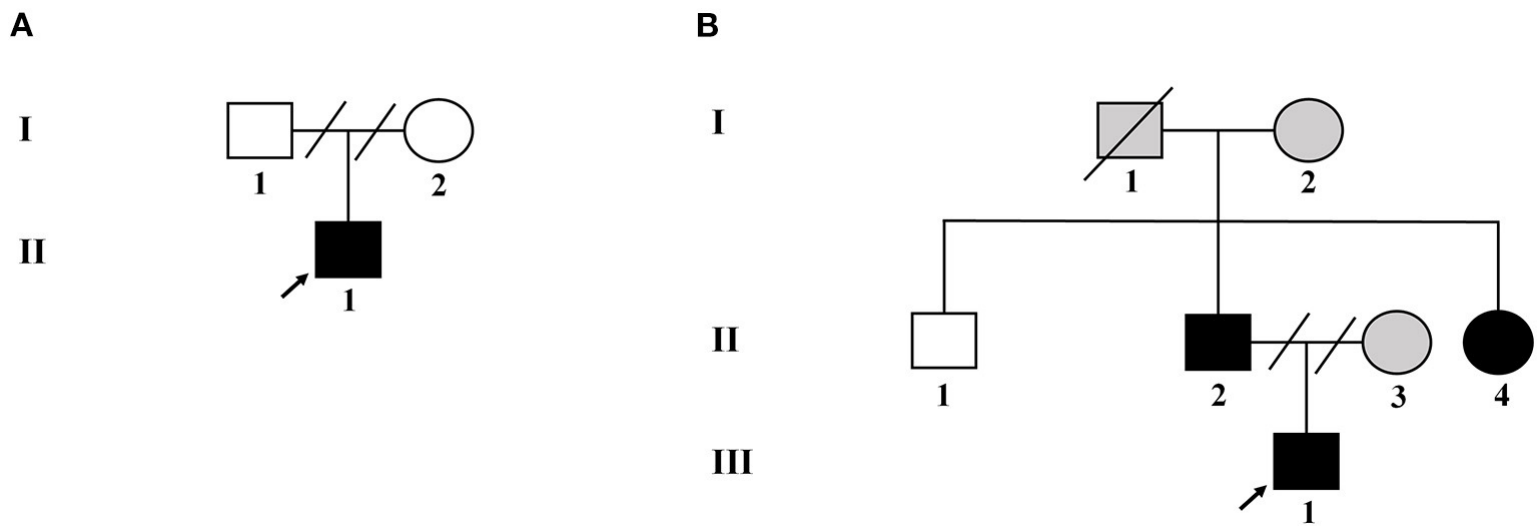
Pedigree of the two unrelated Chinese families with NF-1. **(A)** Pedigree of family 1. **(B)** Pedigree of family 2. Black filled symbols represent affected subjects. Arrowhead indicates the probands. Empty symbols depict unaffected members. Gray filled symbols indicate the subjects without genetic testing. Connecting lines combined with slashes indicate the state of divorce. Diagonal line means decreased subjects.

Two unrelated families, comprising four patients with NF-1 and three unaffected individuals, were recruited for this study. The pedigree chart was shown in Figure 1. Both families had no consanguineous marriages. Patient 1 (proband II-1 in family 1), a 26-year-old man with symptoms of multiple CALM, cutaneous neurofibromas on his chest and back, axillary and inguinal freckling, early-onset hypertension, hypokalemia, and a history of stroke, was referred to the Department of Hypertension in Fuwai Hospital (Beijing, China). Patient 2 (proband III-1 in family 2) was a 14-year-old adolescent boy with CALM and axillary and inguinal freckling who was initially admitted to hospital because of early-onset hypertension and headache. The diagnosis of NF-1 critically depends on the revised diagnosis criteria for NF-1 updated in 2021 (12). The diagnosis of hypertension in adults and adolescents is based on the European Society of Cardiology and the European Society of Hypertension guidelines for the management of arterial hypertension (13). During hospitalization, detailed clinical data, including medical history, physical examination, and laboratory and imaging evaluation, were collected.

This study complied with the principles of the Declaration of Helsinki and was approved by the Ethics Committee of Fuwai Hospital. Informed consent forms were obtained in advance from all the recruited participants.

### Whole-Exome Sequencing and Bioinformatic Analysis

To identify candidate genes and causal variants, whole-exome sequencing was performed on the two probands (II-1 in family 1 and III-1 in family 2). Genomic DNA was extracted from peripheral blood leukocytes from all the participants using a QIA Amp DNA Blood Mini kit (QIAGEN, Hilden, Germany) in accordance with the manufacturer's protocol. The concentration and quality of each DNA samples were evaluated by Thermal Nanodrop 2000 and agarose gel electrophoresis, respectively. To generate the DNA libraries, genomic DNA fragmentation, end repair, adapter ligation, and PCR enrichment were performed according to Illumina protocols. A GenCap exome capture kit (MyGenostics GenCap Enrichment technologies) was used for exome capture. DNA sequencing was performed on the Illumina HiSeq X Ten system. After removing Low-quality reads and adaptor sequences, the Burrows-Wheeler Aligner tool was used to align the clean reads to the human reference genome (UCSC, GRCh37/hg19). Duplicate reads were removed using the Picard software (http://broadinstitute.github.io/picard/). Subsequently, insertions/deletions and single nucleotide polymorphisms were identified using GATK (http://www.broadinstitute.org/gsa/wiki/index.php/Home_Page) and SOAPsnp (http://soap.genomics.org.cn/soapsnp.html). Variants were filtered in multiple databases, including the 1,000 Genomes Project database (http://browser.1000genomes.org/), dbSNP (https://www.ncbi.nlm.nih.gov/snp/), the Human Gene Mutation Database (HGMD, http://www.hgmd.cf.ac.uk/), and Exome Aggregation Consortium (http://exac.broadinstitute.org/). Additional *in silico* analyses were performed using PolyPhen-2 (http://genetics.bwh.harvard.edu/pph2/), SIFT (http://sift.jcvi.org/), and MutationTaster (http://www.mutationtaster.org/) (14). The neurofibromin amino acid sequences from eight different species were aligned using ClustalW (https://www.genome.jp/tools-bin/clustalw). The pathogenicity of potential mutations was evaluated according to the American College of Medical Genetics and Genomics guidelines for the interpretation of sequence variants that were published in 2015 (15).

### Sanger Sequencing Validation

Sanger sequencing was used to confirm the candidate variants detected by the whole-exome sequencing within the two families. Co-segregation analyses were performed in relevant available individuals. The designed primer sequences are listed in Supplementary Table 1 and the sequencing results were read using Chromas software (version 2.22; Technelysium Pty, Ltd.).

### Systematic Review

We performed literature research using “neurofibromatosis type 1,” “von Recklinghausen disease,” “neurofibromatosis-1,” “hypertension,” “elevated blood pressure,” and “blood pressure” in the PubMed and Embase databases with no year limit. Publications that met the following predetermined criteria were excluded: (1) articles were not in English; (2) article types were book section, or review; (3) articles were related to pulmonary, gestational, intracranial, or intraocular hypertension; (4) animal models or fundamental experiments were involved. Only articles with subjects who were general patients with NF-1 were included in the review. Variables including author, years of publication, number of patients with NF-1, method of blood pressure measurement, blood pressure results, secondary causes of hypertension, and complications of NF-1 were extracted from the selected articles by two authors independently.

## Results

### Clinical Manifestations

**Figure 2:**
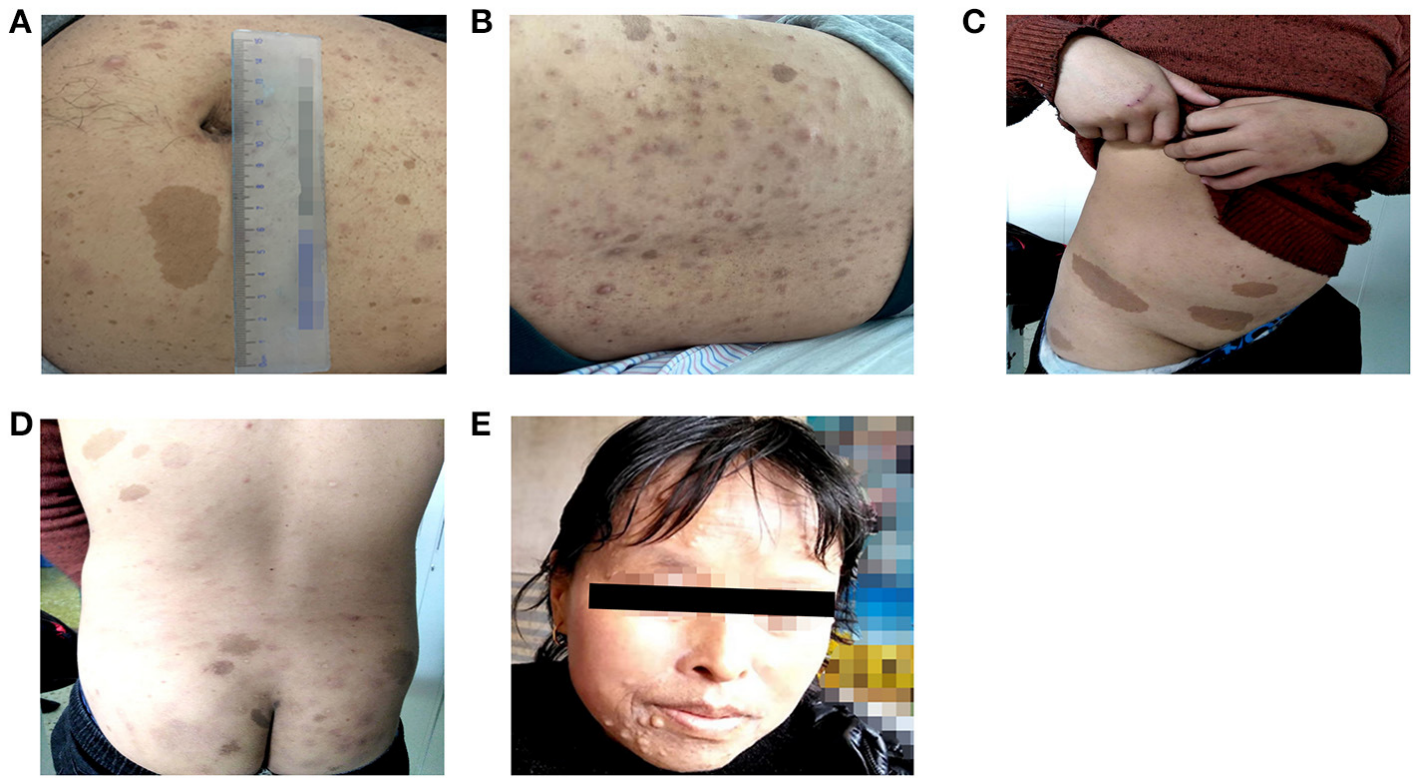
Cutaneous lesions of patients in two unrelated families. **(A,B)** Multiple Café-au-lait macules and cutaneous neurofibromas of patient 1. **(C,D)** Multiple Café-au-lait macules scattered in patient 2. **(E)** Cutaneous neurofibromas of patient 2′ aunt.

Patient 1 was a 26-year-old man (proband II-1 in family 1) who was admitted to the Fuwai Hospital with suspected NF-1 and early-onset hypertension. When he was 6 years old, a mass with a diameter of 7 mm was found on his left palm, which was later confirmed as neurofibroma by pathological biopsy after resection (clinical data not available). Gradually, multiple CALM and cutaneous neurofibromas appeared, distributing mainly on his abdomen and anterior and posterior thorax (Figures 2A,B). When he was 25.5 years old, he presented with syncope and was diagnosed with cerebral hemorrhage. Later, he was found to have hypertension and hypokalemia with no symptoms during his rehabilitation for cerebral hemorrhage in the local hospital and then admitted to the Fuwai Hospital. An echocardiogram and renal artery and aortic computed tomography (CT) did not reveal any abnormalities, whereas adrenal CT showed a space-occupying lesion of the right adrenal gland, which was presumed to be an adrenal neurofibroma. Laboratory examinations including urine free normetanephrine (NMN) and metanephrine (MN) were all normal (NMN 185μg/24h, normal range 28-615μg/24h; MN 149μg/24h, normal range 14-282 μg/24h) except for hypokalemia (3.12 mmol/L, normal range 3.5–5.3 mmol/L) and a slightly elevated plasma renin concentration (66.1 μIU/mL, normal range 4.4–46.1 μIU/mL), ruling out the possibility of pheochromocytoma. Ophthalmologic examination found Lisch nodules in this patient; his parents showed no abnormal clinical symptoms.

Four individuals in family 2 participated in the study. The proband was a 14-year-old adolescent boy (III-1 in family 2) referred to hospital for early-onset hypertension with severe headache. Physical examination found mental retardation (low IQ, attention deficit and hyperactivity disorder), multiple CALM on his neck and trunk, and axillary freckling (Figures 2C,D). Head CT showed no abnormalities and the adrenal CT screen showed that the medial branch of the left adrenal gland was slightly thickened. Laboratory examination including serum electrolytes and blood biochemistry test was normal. Because of economic constraints, the proband's father had refused further examinations. The proband' father (II-2), and aunt (II-4) presented with multiple cutaneous neurofibromas (Figure 2E), and no abnormalities were found in his uncle (II-1). Because the proband's parents were divorced, no related information about the proband's mother (II-3) was obtained.

### Genetic Analysis

**Figure 3:**
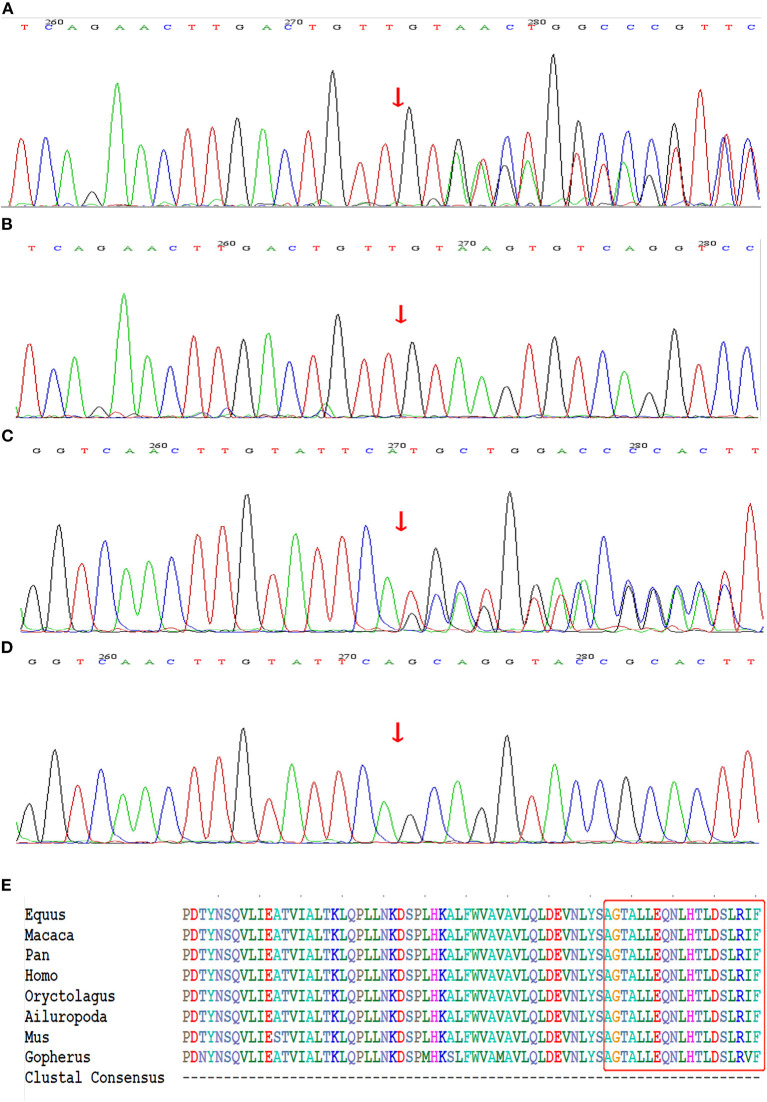
Genetic sequencing results of *NF1* gene. **(A)** The recurrent frame-shift mutation c.6789_6792delTTAC (arrow) found in the index patient of family 1. **(B)** Wild-type found in unaffected subjects of family 1. **(C)** The novel frame-shift mutation c.6934_6936delGCAinsTGCT (arrow) found in affected subjects of family 2. **(D)** Wild-type found in unaffected subject of family 2. **(E)** The mutation region of neurofibromin protein identified in family 2 is highly conserved by analyzing orthologs from 8 various species on CLUSTALW tool.

The whole-exome sequencing of the samples from the two probands yielded approximate 17 GB and 11 GB raw data with more than 99% coverage of the target region. The average sequencing depths were 213 and 123 in proband II-1 (family 1) and III-1 (family 2), respectively, with more than 98% of the bases read at 10 × coverage and 97% bases read at 20 × coverage. Two heterozygous frame-shift mutations, c.6789_6792delTTAC and c.6934_6936delGCAinsTGCT in *NF1* (NM_000267) were supposed as candidate causative variants associated with the phenotypes of probands II-1 (family 1) and III-1 (family 2), respectively. Both variants were predicted to be pathogenic by MutationTaster and have not been reported in the 1,000 Genome Project, ExAC_ALL, or ExAC_EAS databases (Table 1). The results were confirmed by Sanger sequencing (Figures 3A–D). Moreover, the region in which variant c.6934_6936delGCAinsTGCT is located was found to be highly conserved in the CLUSTALW multiple alignment of neurofibromin amino acid sequences from eight different species (Figure 3E). Four NF-1 patients were identified in the two families. In family 1, one recurrent mutation, c.6789_6792delTTAC, was detected in proband II-1. The mutation was not detected in his parents and was considered as a *de novo* variant. In family 2, a novel potential mutation, c.6934_6936delGCAinsTGCT, was detected in the proband (III-1) and another two family members (II-2, and II-4), which co-segregated with the disease presentation in this family.

**Table 1:** Annotations of two candidate variants detected in two NF-1 families.

**Subjects**	**Variation**	**Exon**	**Mutation type**	***De novo***	**Het/Hom**	**Novel mutation**	**Mutation-Taster**	**PhastCons**	**Pathogenicity analysis of ACMG standard**	**Allele frequency of the identified variants in the general population**
1	c.6789_6792delTTAC	45	Deletion	Yes	Het	No	Disease-causing	1	Pathogenic (PVS1 + PM2 + PP4 + PS2)	None
2	c.6934_6936delGCAinsTGCT	46	Deletion-insertion	No	Het	Yes	Disease-causing	1	Pathogenic (PVS1 + PM2 + PP4)	None

*NF-1, neurofibromatosis type 1; het, heterozygote; hom, homozygote; ACMG, American College of Medical Genetics and Genomics; the allele frequency of the identified variants in the general population are queried in the gnomAD database*.

### Systematic Literature Review

**Figure 4:**
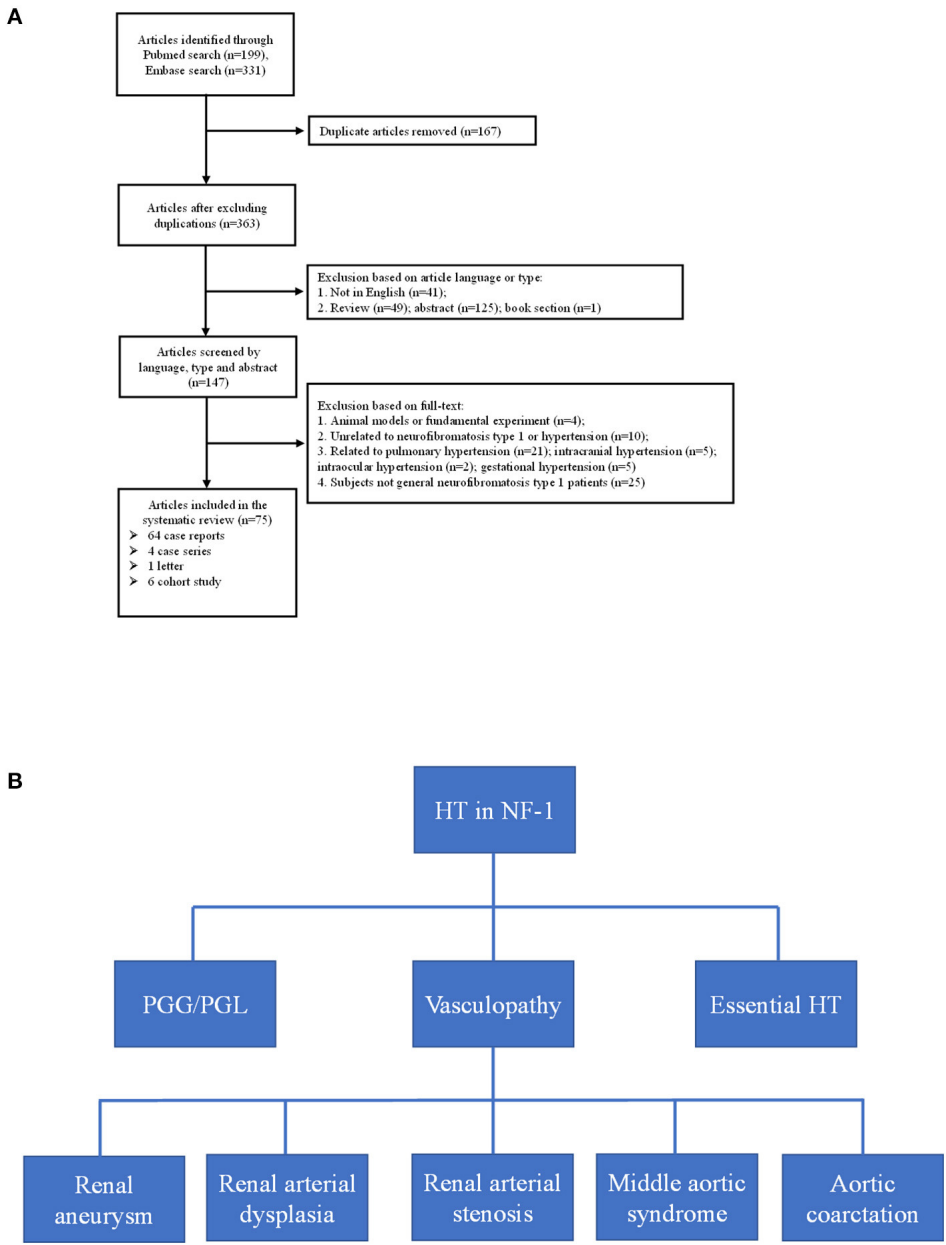
**(A)** Flow-chart of the eligible articles extraction. **(B)** Etiologies of neurofibromatosis type 1 with hypertension. HT, hypertension; PGG/PGL, pheochromocytoma/paraganglioma.

Through the literature search, we retrieved a total of 363 articles after excluding duplications; the selection flowchart is shown in Figure 4A. After reading the title, abstract, or full text, a total of 75 eligible articles were identified, comprising six cohorts from seven articles. It should be noted that the enrolled patients of the two articles published by Tedesco et al. (16, 17) were identical, but they used different blood pressure measurement methods. The variables we analyzed are extracted from the selected articles by two authors independently and the results are listed in Table 2.

**Table 2:** Summary of clinical characteristics of eligible studies included in the literature review.

**References**	**NF-1 patients enrolled**	**% Male**	**Age, years mean ± SD (range)**	**Diagnostic criteria**	**BP measurements**	**BP condition (number, %)**			**Secondary cause of HT**	**Complications of NF-1 associated with HT (patient number)**
						**Normal**	**Pre-HT**	**HT**		
Virdis (18)	57	-	- (range 1.5–23)	NA	OBPM	48/57 (84.2)		9/57 (15.8)^*^	RAS	RAS (2), brain glioma (3)
Dubov (19)	224	53.1	9.1 ± 4.1 (range 2–17)	NIH	OBPM	107/114 (93.9)^*^		7/114 (6.1)^*^ 23/164 (14.0)^#^	Not screened	NA
						141/164 (86.0)^#^				
Lama (20)	69	52.2	11 ± 4 (range 5–25)	NIH	OBPM	48/68 (70.6)^*^	15/68 (22.0)^*^	5/68 (7.4)^*^	Screened, but not found	Plexiform cervical neurofibroma (1), pulmonary valve stenosis (1), secundum atrial septal defect (1), left pulmonary artery stenosis (1), mild mitral regurgitation (1), mild aortic regurgitation (1), atrial septal aneurysm (1), hypertrophic cardiomyopathy (1)
					ABPM	57/68 (84.0)		11/68 (16.0)		
Zinnamosca (21)	48	47.9	40.5 ± 14 (range NA)	NIH	OBPM	37/48 (77.1)^#^		11/48 (22.9)^#^	Pheochromocytoma	Bilateral adrenal pheochromocytoma (1), unilateral adrenal pheochromocytoma (5), autoimmune thyroiditis (1), multiple endocrine neoplasia type 2A (1), Multinodular goiter (1)
Fossali (22)	27	40.7	12.8 ± NA (range 4.2–24)	NA	OBPM	22/27 (81.5)^#^	2/27 (7.4)^#^	3/27 (11.1)^#^	RAS, MAS	Renal artery stenosis in MAS (2), proximal renal artery stenosis (1)
Tedesco (16, 17)	64	53.1	12 ± 4 (range 5–25)	NIH	OBPM	49/64 (76.5)^*^		15/64 (23.4)^*^	RAS	Nephrotic syndrome (1), plexiform cervical neurofibroma (1), RAS (4)
					ABPM	54/64 (84.0)		10/64 (16.0)		

*BP, blood pressure; NF-1, neurofibromatosis type 1; HT, hypertension; OBPM, office blood pressure monitor; ABPM, ambulatory blood pressure monitor; NIH, diagnostic criteria established by National Institutes of Health; RAS, renal artery stenosis; MAS, middle aortic stenosis; NA, not mentioned*;

* *blood pressure measurement elevated at least three different sessions*;

# *blood pressure elevated at once or twice sessions*.

## Discussion

In this study, we identified two frame-shift mutations in *NF1* among four affected individuals from two unrelated Chinese families using whole-exome sequencing. One mutation (c.6789_6792delTTAC) is a *de novo* mutation that has been reported previously; the other mutation (c.6934_6936delGCAinsTGCT) is reported here for the first time, to our knowledge, with evident pathogenicity predicted by the bioinformatic analysis and family co-segregation. Furthermore, by conducting a systematic literature review on patients with NF-1 associated with hypertension, we found that hypertension was a relatively common complication of NF-1, with a prevalence of 6.1–23.4%.

NF-1 is one of the most common genetic disease caused by mutations in *NF1*, which encodes neurofibromin protein. Neurofibromin functions as a RAS-GTPase activating protein that accelerates the conversion of the active form RAS-GTP to the inactive form RAS-GDP (23). Neurofibromin deficiency results in unchecked RAS signaling followed by improper activation of multiple downstream pathways, such as the RAS/mitogen-activated protein kinase (RAS/MAPK), and PI3K-mTOR pathways, leading to unregulated cell survival and proliferation (24).

Because of the large size and complex structure of *NF1*, to date, more than 3710 *NF1* mutations have been collected in the HGMD database but exact mutation hot spots have not been identified. Nearly half of affected individuals reported till now had *de novo* mutations in *NF1* and an absence of a familial NF-1 history, which is what we found for the proband (II-1) of family 1 (25). The mutation c.6789_6792delTTAC detected in family 1 has been reported previously. In 1995, Robinson et al. (26) were the first to identify this small deletion mutation by PCR amplification of specific exon of *NF1* among 92 unrelated patients with NF-1. The c.6789_6792delTTAC mutation caused a frame shift that led to the formation of a premature stop signal five codons downstream, resulting in a truncated protein. Subsequently, several patients carrying the same mutation have been identified, suggesting this 4-bp region of the exon may be prone to mutation (27–29). This might be because the symmetrical sequence tends to form a loop-like structure, leaving an unpaired loop-region, which includes the 4-bp region, more accessible for the generation of deletion variants (26, 30). The novel variant c.6934_6936delGCAinsTGCT identified in family 2 caused the replacement of alanine with cysteine at codon 2,312 and created a frame-shift that led to the formation of a premature stop codon at position 2,318. The CLUSTALW multiple alignment of neurofibromin amino acid sequences from eight different species showed the region in which variant c.6934_6936delGCAinsTGCT is located was highly conserved. Moreover, the MutationTaster prediction together with the evaluation of American College of Medical Genetics and Genomics standards indicted that c.6934_6936delGCAinsTGCT was a disease-causing mutation.

Hypertension is not rare in patients with NF-1 for essential or secondary causes. In our study, both probands had early-onset hypertension. Our literature review found six cohorts from seven articles that considered the prevalence of pre-hypertension and hypertension in patients with NF-1; 7.4–22% and 6.1–23.4% for pre-hypertension and hypertension, respectively (16–22). In five of the studies (16–20, 22), the patients were children and young adults (1.5–25 years) with a high prevalence of early-onset hypertension, suggesting the necessity of regular blood pressure monitor in young patients with NF-1, and the patients in the study conducted by Zinnamosca et al. (21) were middle-aged adults. Dubov et al. (19) found the lowest prevalence of hypertension (6.1%) by measuring office blood pressure of 114 patients with NF-1, whereas Tedesco et al. (17) found the highest prevalence (23.4%) using the same method and criteria in a different cohort of 64 patients with NF-1. The prevalence identified by Virdis et al. (18) using office blood pressure measurement was 15.7%, which was in the middle of the above two study. The hypertension prevalence reported by Tedesco et al. (16) using ambulatory blood pressure (ABPM) is consistent with that of the Lama et al. (20) study (approximately 16.0%) in which ABPM also was used. The differences of hypertension prevalence in these studies may be due not only to the different NF-1 populations but also to the different methodologies used to monitor blood pressure. Generally, ABPM is considered to be a reliable and stable diagnostic procedure to detect hypertension (31). Early diagnosis and understanding the etiologies of hypertension in patients with NF-1 is a prerequisite for blood pressure control, in avoid of poor outcome including hemorrhagic stroke, retinal arterial microaneurysms, end stage renal disease and so on (32, 33). After summarizing the literature of eligible cohorts, case reports, case series and so on, the secondary causes of hypertension basically include renal aneurysm, renal arterial dysplasia, renal artery stenosis, aortic coarctation, pheochromocytoma, and middle aortic syndrome (Figure 4B). In our study, the possibility of secondary hypertension in the two probands was mostly ruled out by the imaging and laboratory examination, and the early-onset hypertension was considered to be possibly associated with low-grade vasculopathy (20).

NF-1 disease demonstrates with complete penetrance but high phenotypic variability is present even in families that carry the same mutation (34), as was reflected in our study. In family 2, the types and degrees of clinical manifestations of the affected members varied. Mental retardation was found only in the proband, and the other members with NF-1 showed normal behavior. Moreover, the proband's aunt and father had cutaneous neurofibroma, but the proband did not. NF-1 is an age-dependent progressive disorder, and therefore the phenotypic variability may be partly explained by age differences among patients (35). Several large familial NF-1 studies revealed that unlinked modifier genes may influence the expression of the disease (36, 37). Recently, using a systems biology strategy, Kowalski et al. (38) identified 10 candidate modifier genes related to the NF-1 phenotype, namely *AKT1, BRAF, EGFR, LIMK1, PAK1, PTEN, RAF1, SDC2, SMARCA4*, and *VCP*. What's more, D'Amico et al. (39) identified that gain-of-function and hypomorphic variants in *PTPN11*, a positive regulator of RAS, have been shown to worsen and mitigate, respectively, the severity of the NF1 phenotype. Besides, the allelic heterogeneity of constitutional *NF1* mutations, environmental factors may also be associated with phenotypic variability (40, 41). Overall, the exact mechanism of clinical variability in NF-1 remains unclear and may involve a combination of multiple factors.

Because of the wide mutation spectrum and variable phenotypes expressed, only a few reliable genotype–phenotype correlations have been reported in NF-1 so far. Patients with *NF1* microdeletions tended to develop more severe phenotypes that manifest as learning disabilities, facial dysmorphism, cardiovascular anomalies, increased numbers of neurofibromas, and malignant peripheral nerve sheath tumors (42, 43). Among the intragenic *NF1* mutations, the deletion mutation p.Met922del in exon 17 has been correlated with a mild phenotype of typical pigment lesions (CALM or freckling), learning difficulties, and lacking forms of neurofibromas (44, 45). Other intragenic missense mutations that influenced p.Arg1809 have also been correlated with mild presentation characterized by a pigmentary phenotype, Noonan-like features, pulmonic stenosis, stature dysplasia, and absence of visible neurofibromas (46, 47). Kang et al. (48) studied patients with different mutation types and found that patients with truncating/splicing mutations and large deletions tended to present with a more severe phenotype and earlier onset age than those with missense types. Recently, Scala et al. (49) identified that frameshift variants and whole gene deletion were correlated with skeletal abnormalities, whereas neurofibromas were negatively associated with missense variants. Moreover, they found that the presence of structural brain alterations was associated with c.3721C>T variant, whereas Lisch nodules and endocrinological disorders were more common observed in NF-1 patients with c.6855C>A variant. However, apart from the phenotypic diversity in family 2, because the recurrent mutation detected in proband II-1 in family 1 was *de novo* and the clinical information of previously reported patients with the same mutation was limited, we failed to construct genotype–phenotype correlations in this study.

Because patients with NF-1 are at increased multiple risk of developing cerebrovascular diseases and benign or malignant tumors, early identification can help targeted examination, multidisciplinary management, and long-term clinical monitoring (8, 50). Genetic testing can provide valuable clues to NF-1 based on molecular diagnosis, especially for young patients with no positive family history or typical symptoms who do not meet the criteria for a clinical diagnosis (50). It is also necessary to conduct genetic counseling for first-degree or second-degree relatives of affected patients. Next-generation sequencing technologies have helped make the molecular analysis of mutations in the large wide-spectrum *NF1* gene more cost/time-effective and highly precise. Furthermore, regular multi-systematic follow-ups should be offered to affected patients to prevent the occurrence of fatal complications.

## Conclusion

In summary, we identified one recurrent frame-shift mutation c.6789_6792delTTAC and one novel frame-shift mutation c.6934_6936delGCAinsTGCT in two unrelated families with NF-1 from China. Genetic testing is a useful and precise method for diagnosing the disease at the molecular level in spite of phenotypic variability. Hypertension is not a rare complication of NF-1, and it was seen in both probands in this study. Routine screening for hypertension in patients with NF-1, especially children and adolescents, is important to avoid serious cardiovascular events. Given the phenotypic variability, more research is needed to unravel the mechanisms that influence how genetic and other factors determine the NF-1 phenotypes.

## Data Availability Statement

The datasets for this article are not publicly available due to concerns regarding participant/patient anonymity. Requests to access the datasets should be directed to the corresponding author.

## Ethics Statement

The studies involving human participants were reviewed and approved by the Ethics Committee of Fuwai Hospital. Written informed consent to participate in this study was provided by the participant's legal guardian/next of kin.

## Author Contributions

Y-TL, YX, and X-LZ designed, supervised the study, and modified the manuscript. Y-TL, DZ, and X-CL collected samples and clinical information. Y-TL, X-QD, and Q-YZ performed the experiments. Y-TL and PF performed the data analysis. Y-TL and DZ wrote the manuscript. All authors reviewed this work.

## Funding

This work was supported by the Non-profit Central Research Institute Fund of Chinese Academy of Medical Sciences (2019XK320057), the National Key Research and Development Program of China (2016YFC1300100).

## Conflict of Interest

The authors declare that the research was conducted in the absence of any commercial or financial relationships that could be construed as a potential conflict of interest.

## Publisher's Note

All claims expressed in this article are solely those of the authors and do not necessarily represent those of their affiliated organizations, or those of the publisher, the editors and the reviewers. Any product that may be evaluated in this article, or claim that may be made by its manufacturer, is not guaranteed or endorsed by the publisher.

## References

[1] Hirbe AC Gutmann DH. Neurofibromatosis type 1: a multidisciplinary approach to care. Lancet Neurol. (2014) 13:834–43. 10.1016/S1474-4422(14)70063-8 25030515

[2] Williams VC Lucas J Babcock MA Gutmann DH Korf B Maria BL. Neurofibromatosis type 1 revisited. Pediatrics. (2009) 123:124–33. 10.1542/peds.2007-3204 19117870

[3] Gottfried ON Viskochil DH Couldwell WT. Neurofibromatosis Type 1 and tumorigenesis: molecular mechanisms and therapeutic implications. Neurosurg Focus. (2010) 28:E8. 10.3171/2009.11.FOCUS09221 20043723

[4] Delis KT Gloviczki P. Neurofibromatosis type 1: from presentation and diagnosis to vascular and endovascular therapy. Perspect Vasc Surg Endovasc Ther. (2006) 18:226–37. 10.1177/1531003506296488 17172538

[5] Armand ML Taieb C Bourgeois A Bourlier M Bennani M Bodemer C. Burden of adult neurofibromatosis 1: development and validation of a burden assessment tool. Orphanet J Rare Dis. (2019) 14:94. 10.1186/s13023-019-1067-8 31053133 PMC6500066

[6] Bergqvist C Servy A Valeyrie-Allanore L Ferkal S Combemale P Wolkenstein P. Neurofibromatosis 1 French national guidelines based on an extensive literature review since 1966. Orphanet J Rare Dis. (2020) 15:37. 10.1186/s13023-020-1310-3 32014052 PMC6998847

[7] Sivasubramanian R Meyers KE. Hypertension in children and adolescents with turner syndrome (TS), neurofibromatosis 1 (NF1), and williams syndrome (WS). Curr Hypertens Rep. (2021) 23:18. 10.1007/s11906-021-01136-7 33779870

[8] Terry AR Jordan JT Schwamm L Plotkin SR. Increased risk of cerebrovascular disease among patients with neurofibromatosis type 1: population-based approach. Stroke. (2016) 47:60–5. 10.1161/STROKEAHA.115.011406 26645253

[9] Friedman JM Arbiser J Epstein JA Gutmann DH Huot SJ Lin AE. Cardiovascular disease in neurofibromatosis 1: report of the NF1 Cardiovascular Task Force. Genet Med. (2002) 4:105–11. 10.1097/00125817-200205000-00002 12180143

[10] Kim SS Stein DR Ferguson MA Porras D Chaudry G Singh MN. Surgical management of pediatric renovascular hypertension and midaortic syndrome at a single-center multidisciplinary program. J Vasc Surg. (2020) 74:79–89. 10.1016/j.jvs.2020.12.053 33340698

[11] Moramarco J El Ghorayeb N Dumas N Nolet S Boulanger L Burnichon N. Pheochromocytomas are diagnosed incidentally and at older age in neurofibromatosis type 1. Clin Endocrinol. (2017) 86:332–9. 10.1111/cen.13265 27787920

[12] Legius E Messiaen L Wolkenstein P Pancza P Avery RA Berman Y. Revised diagnostic criteria for neurofibromatosis type 1 and Legius syndrome: an international consensus recommendation. Genet Med. (2021) 23:1506–13. 10.1038/s41436-021-01170-5 34012067 PMC8354850

[13] Williams B Mancia G Spiering W Agabiti Rosei E Azizi M Burnier M. 2018 ESC/ESH Guidelines for the management of arterial hypertension. Eur Heart J. (2018) 39:3021–104. 10.1097/HJH.0000000000001940 30165516

[14] Schwarz JM Rodelsperger C Schuelke M Seelow D. MutationTaster evaluates disease-causing potential of sequence alterations. Nat Methods. (2010) 7:575–6. 10.1038/nmeth0810-575 20676075

[15] Richards S Aziz N Bale S Bick D Das S Gastier-Foster J. Standards and guidelines for the interpretation of sequence variants: a joint consensus recommendation of the American college of medical genetics and genomics and the association for molecular pathology. Genet Med. (2015) 17:405–24. 10.1038/gim.2015.30 25741868 PMC4544753

[16] Tedesco MA Di Salvo G Ratti G Natale F Calabrese E Grassia C. Arterial distensibility and ambulatory blood pressure monitoring in young patients with neurofibromatosis type 1. Am J Hypertens. (2001) 14:559–66. 10.1016/S0895-7061(00)01303-0 11411736

[17] Tedesco MA Ratti G Di Salvo G Martiniello AR Limongelli G Grieco M. Noninvasive evaluation of arterial abnormalities in young patients with neurofibromatosis type 1. Angiology. (2000) 51:733–41. 10.1177/000331970005100905 10999614

[18] Virdis R Balestrazzi P Zampolli M Donadio A Street M Lorenzetti E. Hypertension in children with neurofibromatosis. J Hum Hypertens. (1994) 8:395–7. 8064789

[19] Dubov T Toledano-Alhadef H Chernin G Constantini S Cleper R Ben-Shachar S. High prevalence of elevated blood pressure among children with neurofibromatosis type 1. Pediatr Nephrol. (2016) 31:131–6. 10.1007/s00467-015-3191-6 26314566

[20] Lama G Graziano L Calabrese E Grassia C Rambaldi PF Cioce F. Blood pressure and cardiovascular involvement in children with neurofibromatosis type1. Pediatr Nephrol. (2004) 19:413–8. 10.1007/s00467-003-1397-5 14991390

[21] Zinnamosca L Petramala L Cotesta D Marinelli C Schina M Cianci R. Neurofibromatosis type 1 (NF1) and pheochromocytoma: prevalence, clinical and cardiovascular aspects. Arch Dermatol Res. (2011) 303:317–25. 10.1007/s00403-010-1090-z 21042801

[22] Fossali E Signorini E Intermite RC Casalini E Lovaria A Maninetti MM. Renovascular disease and hypertension in children with neurofibromatosis. Pediatr nephrol. (2000) 14:806–10. 10.1007/s004679900260 10955932

[23] Bajaj A Li QF Zheng Q Pumiglia K. Loss of NF1 expression in human endothelial cells promotes autonomous proliferation and altered vascular morphogenesis. PLoS ONE. (2012) 7:e49222. 10.1371/journal.pone.0049222 23145129 PMC3492274

[24] Nix JS Blakeley J Rodriguez FJ. An update on the central nervous system manifestations of neurofibromatosis type 1. Acta Neuropathol. (2020) 139:625–41. 10.1007/s00401-019-02002-2 30963251 PMC6819239

[25] Evans DG Howard E Giblin C Clancy T Spencer H Huson SM. Birth incidence and prevalence of tumor-prone syndromes: estimates from a UK family genetic register service. Am J Med Genet. (2010) 152A:327–32. 10.1002/ajmg.a.33139 20082463

[26] Robinson PN Boddrich A Peters H Tinschert S Buske A Kaufmann D. Two recurrent nonsense mutations and a 4 bp deletion in a quasi-symmetric element in exon 37 of the NF1 gene. Hum Genet. (1995) 96:95–8. 10.1007/BF00214193 7607663

[27] Boddrich A Robinson PN Schulke M Buske A Tinschert S Nurnberg P. New evidence for a mutation hotspot in exon 37 of the NF1 gene. Hum Mutat. (1997) 9:374–7. 10.1002/(SICI)1098-1004(1997)9:4 <374::AID-HUMU15>3.0.CO;2-# 9101303

[28] Fahsold R Hoffmeyer S Mischung C Gille C Ehlers C Kucukceylan N. Minor lesion mutational spectrum of the entire NF1 gene does not explain its high mutability but points to a functional domain upstream of the GAP-related domain. Am J Hum Genet. (2000) 66:790–818. 10.1086/302809 10712197 PMC1288164

[29] Origone P De Luca A Bellini C Buccino A Mingarelli R Costabel S. Ten novel mutations in the human neurofibromatosis type 1 (NF1) gene in Italian patients. Hum Mutat. (2002) 20:74–5. 10.1002/humu.9039 12112660

[30] Ekvall S Sjors K Jonzon A Vihinen M Anneren G Bondeson ML. Novel association of neurofibromatosis type 1-causing mutations in families with neurofibromatosis-Noonan syndrome. Am J Med Genet. (2014) 164A:579–87. 10.1002/ajmg.a.36313 24357598

[31] Hermida RC Mojon A Fernandez JR Otero A Crespo JJ Dominguez-Sardina M. Ambulatory blood pressure monitoring-based definition of true arterial hypertension. Minerva Med. (2020) 111:573–88. 10.23736/S0026-4806.20.06834-2 32700870

[32] Faris M Baliss M Coni R Nambudiri V. Severe hypertension leading to hemorrhagic stroke in neurofibromatosis type 1. Cureus. (2021) 13:e14658. 10.7759/cureus.14658 33907652 PMC8066756

[33] Lu J Liu H Zhang L Ma L Zhou H. Corkscrew retinal vessels and retinal arterial macroaneurysm in a patient with neurofibromatosis type 1: a case report. Medicine. (2018) 97:e11497. 10.1097/MD.0000000000011497 30045273 PMC6078759

[34] Shofty B Constantini S Ben-Shachar S. Advances in molecular diagnosis of neurofibromatosis type 1. Semin Pediatr Neurol. (2015) 22:234–9. 10.1016/j.spen.2015.10.007 26706011

[35] Pasmant E Vidaud M Vidaud D Wolkenstein P. Neurofibromatosis type 1: from genotype to phenotype. J Med Genet. (2012) 49:483–9. 10.1136/jmedgenet-2012-100978 22889851

[36] Sabbagh A Pasmant E Laurendeau I Parfait B Barbarot S Guillot B. Unravelling the genetic basis of variable clinical expression in neurofibromatosis 1. Hum Mol Genet. (2009) 18:2768–78. 10.1093/hmg/ddp212 19417008 PMC2722187

[37] Szudek J Joe H Friedman JM. Analysis of intrafamilial phenotypic variation in neurofibromatosis 1 (NF1). Genet Epidemiol. (2002) 23:150–64. 10.1002/gepi.1129 12214308

[38] Kowalski TW Reis LB Finger Andreis T Ashton-Prolla P Rosset C. Systems biology approaches reveal potential phenotype-modifier genes in neurofibromatosis Type 1. Cancers. (2020) 12:2416. 10.3390/cancers12092416 32858845 PMC7565824

[39] D'Amico A Rosano C Pannone L Pinna V Assunto A Motta M. Clinical variability of neurofibromatosis 1: a modifying role of cooccurring PTPN11 variants and atypical brain MRI findings. Clin Genet. (2021) 100:563–72. 10.1111/cge.14040 34346503

[40] Rasmussen SA Friedman JM. NF1 gene and neurofibromatosis 1. Am J Epidemiol. (2000) 151:33–40. 10.1093/oxfordjournals.aje.a010118 10625171

[41] Sabbagh A Pasmant E Imbard A Luscan A Soares M Blanche H. NF1 molecular characterization and neurofibromatosis type I genotype-phenotype correlation: the French experience. Hum Mutat. (2013) 34:1510–8. 10.1002/humu.22392 23913538

[42] Pasmant E Sabbagh A Spurlock G Laurendeau I Grillo E Hamel MJ. NF1 microdeletions in neurofibromatosis type 1: from genotype to phenotype. Hum Mutat. (2010) 31:E1506–18. 10.1002/humu.21271 20513137

[43] Kehrer-Sawatzki H Mautner VF Cooper DN. Emerging genotype-phenotype relationships in patients with large NF1 deletions. Hum Genet. (2017) 136:349–76. 10.1007/s00439-017-1766-y 28213670 PMC5370280

[44] Koczkowska M Callens T Gomes A Sharp A Chen Y Hicks AD. Expanding the clinical phenotype of individuals with a 3-bp in-frame deletion of the NF1 gene (c.2970_2972del): an update of genotype-phenotype correlation. Genet Med. (2019) 21:867–76. 30190611 10.1038/s41436-018-0269-0 PMC6752285

[45] Upadhyaya M Huson SM Davies M Thomas N Chuzhanova N Giovannini S. An absence of cutaneous neurofibromas associated with a 3-bp inframe deletion in exon 17 of the NF1 gene (c.2970-2972 delAAT): evidence of a clinically significant NF1 genotype-phenotype correlation. Am J Hum Genet. (2007) 80:140–51. 10.1086/510781 17160901 PMC1785321

[46] Pinna V Lanari V Daniele P Consoli F Agolini E Margiotti K. p.Arg1809Cys substitution in neurofibromin is associated with a distinctive NF1 phenotype without neurofibromas. Eur J Hum Genet. (2015) 23:1068–71. 10.1038/ejhg.2014.243 25370043 PMC4795103

[47] Rojnueangnit K Xie J Gomes A Sharp A Callens T Chen Y. High incidence of noonan syndrome features including short stature and pulmonic stenosis in patients carrying NF1 missense mutations affecting p.Arg1809: genotype-phenotype correlation. Hum Mutat. (2015) 36:1052–63. 10.1002/humu.22832 26178382 PMC5049609

[48] Kang E Kim YM Seo GH Oh A Yoon HM Ra YS. Phenotype categorization of neurofibromatosis type I and correlation to NF1 mutation types. J Hum Genet. (2020) 65:79–89. 10.1038/s10038-019-0695-0 31776437

[49] Scala M Schiavetti I Madia F Chelleri C Piccolo G Accogli A. Genotype-phenotype correlations in neurofibromatosis type 1: a single-center cohort study. Cancers. (2021) 13:1879. 10.3390/cancers13081879 33919865 PMC8070780

[50] Gutmann DH Ferner RE Listernick RH Korf BR Wolters PL Johnson KJ. Neurofibromatosis type 1. Nat Rev Dis Primers. (2017) 3:17004. 10.1038/nrdp.2017.4 28230061

