# Supplementary material for: Identification of NF1 Frameshift Variants in Two Chinese Families With Neurofibromatosis Type 1 and Early-Onset Hypertension
Source: Front Pediatr. 2021 Dec 20;9:785982. doi: 10.3389/fped.2021.785982 (PMC8721095; doi:10.3389/fped.2021.785982)
Supplement: Supplementary file 1 [file Table_1.DOCX]

Table 1. Oligonucleotide primers (5’ to 3’) used for PCR amplification of *NF1* gene

| Name | Forward | Reverse | Annealing temperature (℃) | Product size (bp) |
| --- | --- | --- | --- | --- |
| NF-1 E37 | TCCTGAATTCATTCCGAGATTC | TCTTTCCTTTAGCACTGATGAGAC | 59.8 | 427 |
| NF-1 E38 | GGAGAAGTCAAAGGAGGTGC | GCAACAAGAAAAGATGGAAGAGT | 58.9 | 433 |
